# Efficient biosynthesis of exopolysaccharide in *Candida glabrata* by a fed-batch culture

**DOI:** 10.3389/fbioe.2022.987796

**Published:** 2022-09-02

**Authors:** Sha Xu, Jinke Xu, Weizhu Zeng, Xiaoyu Shan, Jingwen Zhou

**Affiliations:** ^1^ National Engineering Research Center for Cereal Fermentation and Food Biomanufacturing, Jiangnan University, Wuxi, China; ^2^ School of Biotechnology and Key Laboratory of Industrial Biotechnology, Ministry of Education, Jiangnan University, Wuxi, China; ^3^ Jiangsu Provisional Research Center for Bioactive Product Processing Technology, Jiangnan University, Wuxi, China; ^4^ Science Center for Future Foods, Jiangnan University, Wuxi, China

**Keywords:** *Candida glabrata*, exopolysaccharide, medium composition, fed-batch fermentation, overproduction

## Abstract

Polysaccharides are important natural biomacromolecules. In particular, microbial exopolysaccharides have received much attention. They are produced by a variety of microorganisms, and they are widely used in the food, pharmaceutical, and chemical industries. The *Candida glabrata* mutant 4-C10, which has the capacity to produce exopolysaccharide, was previously obtained by random mutagenesis. In this study we aimed to further enhance exopolysaccharide production by systemic fermentation optimization. By single factor optimization and orthogonal design optimization in shaking flasks, an optimal fermentation medium composition was obtained. By optimizing agitation speed, aeration rate, and fed-batch fermentation mode, 118.6 g L^−1^ of exopolysaccharide was obtained by a constant rate feeding fermentation mode, with a glucose yield of 0.62 g g^−1^ and a productivity of 1.24 g L^−1^ h^−1^. Scaling up the established fermentation mode to a 15-L fermenter led to an exopolysaccharide yield of 113.8 g L^−1^, with a glucose yield of 0.60 g g^−1^ and a productivity of 1.29 g L^−1^ h^−1^.

## Introduction

Polysaccharides are important natural biomacromolecules. They are widely present in microorganisms, plants, and animals (Liao et al., 2022; Rajoka et al., 2022). Because of the unique physical and chemical characteristics, such as degradability, bioactivity, nontoxicity, and biocompatibility, polysaccharides are widely applied in the food, pharmaceutical, and chemical industries, where they serve as texture enhancers, stabilizers, gelatinizing agents, emulsifiers, drug carriers, etc. (Kokoulin et al., 2021; Yang et al., 2021; Zhang et al., 2022). Recently, microbial polysaccharides have attracted extensive attention from researchers for their anti-tumor, anti-oxidation, hypoglycemic, and immune-enhancing activities (Saadat et al., 2019; Zeng Y. J. et al., 2019). Microbial polysaccharides are traditionally divided into three types: intracellular polysaccharides, cell wall polysaccharides, and secreted exopolysaccharide (Nachtigall et al., 2020; Tian et al., 2021). In particular, exopolysaccharides have broad application prospects and have attracted increasing attention because of advantages such as relatively high titer, simple separation, and easy large-scale production (Zeng Y. J. et al., 2019).

Exopolysaccharide could be synthesized by various microorganisms, including bacteria, archaea, yeast, fungi, and microalgae (Nambiar et al., 2018; Schmid, 2018). Generally, exopolysaccharides with various structures and high molecular weights are produced and released into the external environment during the process of cell growth or in response to changes in the surrounding environments, such as pH, temperature, ionic strength, and nutritional content (Ayyash et al., 2020; Gan et al., 2020). With the increasing demand for natural biopolymers for diverse industrial applications, various types of exopolysaccharides from different microbial species were investigated and applied (Becker, 2015; Schmid, 2018); for example, hyaluronic acid produced by *Streptococcus zooepidemicus* (Mohan et al., 2022), xanthan gum produced by *Xanthomonas campestris* (Wang Z. C. et al., 2016), and scleroglucan produced by *Sclerotium rolfsii* (Tan et al., 2019). Some kinds of exopolysaccharides have also been isolated from different yeast species, such as *Rhodotorula mucilaginosa* (Ma et al., 2018) and *Zygosaccharomyces rouxii* (Zhang et al., 2022).

*Candida glabrata* is a classical nonconventional yeast and also a multi-vitamin auxotrophic yeast (Raschmanova et al., 2018). Due to the easy cultivation process, the simple culture medium, and the possibility to use renewable resources, *C. glabrata* has become the dominant producer for the important chemical compound pyruvic acid with the microbiological fermentation route (Luo et al., 2019; Guo et al., 2020a). To enhance the production of pyruvic acid, several strategies have been tried, including selecting high-producing strains combined with high-throughput screening and mutagenesis approaches, metabolic engineering to strengthen the synthesis pathway and extracellular transport, and systematic optimization of the fermentation process (Luo et al., 2018; Guo et al., 2020a; Luo et al., 2020). Interestingly, the *C. glabrata* mutant strain 4-C10, which has the capacity to accumulate exopolysaccharide, was obtained by screening for high pyruvic acid production with random mutagenesis in our previous work. The monosaccharide components and formation mechanism of the exopolysaccharide were also analyzed. By knocking out the genes involved in exopolysaccharide synthesis, the pyruvic acid production was enhanced, while the exopolysaccharide accumulation was decreased (Luo et al., 2017a; Luo et al., 2017b).

To further enhance the production of exopolysaccharide with the obtained mutant, *C. glabrata* 4-C10, a systematic optimization of the fermentation process should be conducted. In the present study, based on the employed fermentation medium compositions for pyruvic acid production, suitable fermentation medium compositions for exopolysaccharide production were first obtained with single factor optimization and orthogonal design optimization at the shaking flask level. Then, the effects of agitation speed and aeration rate on exopolysaccharide accumulation were investigated in a 1-L fermenter, and a constant rate feeding fermentation mode was established. The production of exopolysaccharide reached 118.6 g L^−1^. Finally, the established constant rate feeding fermentation mode was scaled up to a 15-L fermenter. The exopolysaccharide production and the product yield were basically stable, laying a foundation for industrial production.

## Materials and methods

### Microorganisms

The producer, *C. glabrata* 4-C10 (CCTCC M2017047), used in this research is a four-vitamin auxotrophic strain (thiamine, biotin, niacin, and pyridoxine), which was previously obtained by random mutagenesis in *C. glabrata* (CCTCC M202019) (Luo et al., 2017a; Luo et al., 2017b).

### Medium

The medium for slant and seeds were consisted of (gL^−1^): glucose 30, plant protein extract 10, KH_2_PO_4_ 1.0 and MgSO_4_·7H_2_O 0.5. Additionally, 20 g L^−1^ of agar was needed to add in the slant medium. The initial fermentation medium consisted of (gL^−1^): glucose 120, urea 3.84, KH_2_PO_4_ 2.0, MgSO_4_·7H_2_O 0.8, CH_3_COONa 3, CaCO_3_ 40. The trace element mixture (10 ml L^−1^) and vitamin mixture (6 ml L^−1^) were added separately. The optimized fermentation medium contained (gL^−1^): glucose 150, urea 5.0, KH_2_PO_4_ 3.0, MgSO_4_·7H_2_O 0.9, CH_3_COONa 3, CaCO_3_ 40. The trace element mixture (10 ml L^−1^) and vitamin mixture (6 ml L^−1^) were also added separately. The trace element mixture was dissolved with 2 M of HCl, consisting of (gL^−1^): MnCl_2_·4H_2_O 12, FeSO_4_·7H_2_O 2, CaCl_2_·2H_2_O 2, CuSO_4_·5H_2_O 0.05, ZnCl_2_ 0.5. The vitamin mixture was dissolved with 2 M of HCl, consisting of (gL^−1^): biotin 0.004, thiamine 0.00075, pyridoxine 0.04, niacin 0.8 (Luo et al., 2017a).

### Culture condition

*C. glabrata* 4-C10 was incubated in seed culture medium at 30°C for 16 h. The seed cultures and fermentation cultivation were implemented in 500 ml shaking flasks consisting of 50 ml of culture medium at 30°C on a reciprocal shaker at 220 rmin^−1^ (Zhichu, Shanghai, China). Batch fermentation and fed-batch fermentation were performed in a 1-L parallel bioreactor (T&J Bio-engineering, Shanghai, China) with a 0.6-L working volume. The agitation speed, aeration rate, and fed-batch mode were adjusted (see Results and discussion section). Fermentation was conducted in a 15-L bioreactor (T&J Bio-engineering, Shanghai, China) containing a 12-L working volume with an agitation speed of 500 rmin^−1^, 1.0 vvm, and a pressure of 0.035 MPa. The pH was automatically kept at 5.5 by adding 8 M of NaOH. The inoculation size was 10% (v/v) and all cultivations were carried out at 30°C (Luo et al., 2017a; Guo et al., 2020b).

### Analytical method

Determination of biomass: The different samples taken in the fermentation process were all diluted to a certain concentration with 2 M HCl. The optical density (OD) of the diluted fermentation broth was detected using a Biospe-1601 spectrophotometer (Shimadzu Co., Kyoto, Japan) at 660 nm. The dry cell weight (DCW) was calculated with the relationship DCW = 0.23×OD_660_. (Luo et al., 2020).

Determination of glucose and pyruvic acid: The glucose was detected with a glucose-lactate biosensor (Sieman Technology, Shenzhen, China), and the pyruvic acid were detected by high-performance liquid chromatography (HPLC, Agilent 1260, CA, United States) with a UV detector at 210 nm with an Aminex HPX-87H column (Bio-Rad, CA, United States). The specific detection conditions were as follows: an injection volume of 10 μl, a mobile phase of 5 mM H_2_SO_4_, a flow rate of 0.5 ml min^−1^, and a column temperature of 40°C (Zhou et al., 2009).

Determination of the concentration of exopolysaccharide: The different samples of fermentation broths were centrifuged at 5000 × *g* for 15 min. Then, two volumes of precooled ethanol (4°C) were added to the obtained supernatant. After 1 h, the precipitate was centrifuged at 5000 × *g* for 15 min. The exopolysaccharide concentration was determined after the precipitate was freeze-dried to a constant weight (Luo et al., 2017a).

### Statistical analysis

All fermentation processes were carried out in triplicate and the results were presented as mean values. The orthogonal experiment was designed by Design Expert and the experimental data were analyzed by Origin 2019b.

## Results and discussion

### Effects of medium components on exopolysaccharide accumulation

For the biosynthesis of specific target metabolites by microbial fermentation, the composition of fermentation medium is usually the preferred optimization factor to enhance the production during the fermentation process (Xin et al., 2017; Wagh et al., 2022). For pyruvic acid production, the optimization of fermentation medium composition and process control was systematically conducted, and significant enhancements in titer and productivity was obtained (Xu et al., 2009; Guo et al., 2020b). For exopolysaccharide production, the optimal addition of vitamin mixture has also been determined (Luo et al., 2017a). To further enhance the exopolysaccharide accumulation, the concentrations of some important components of the medium could be investigated.

**FIGURE 1:**
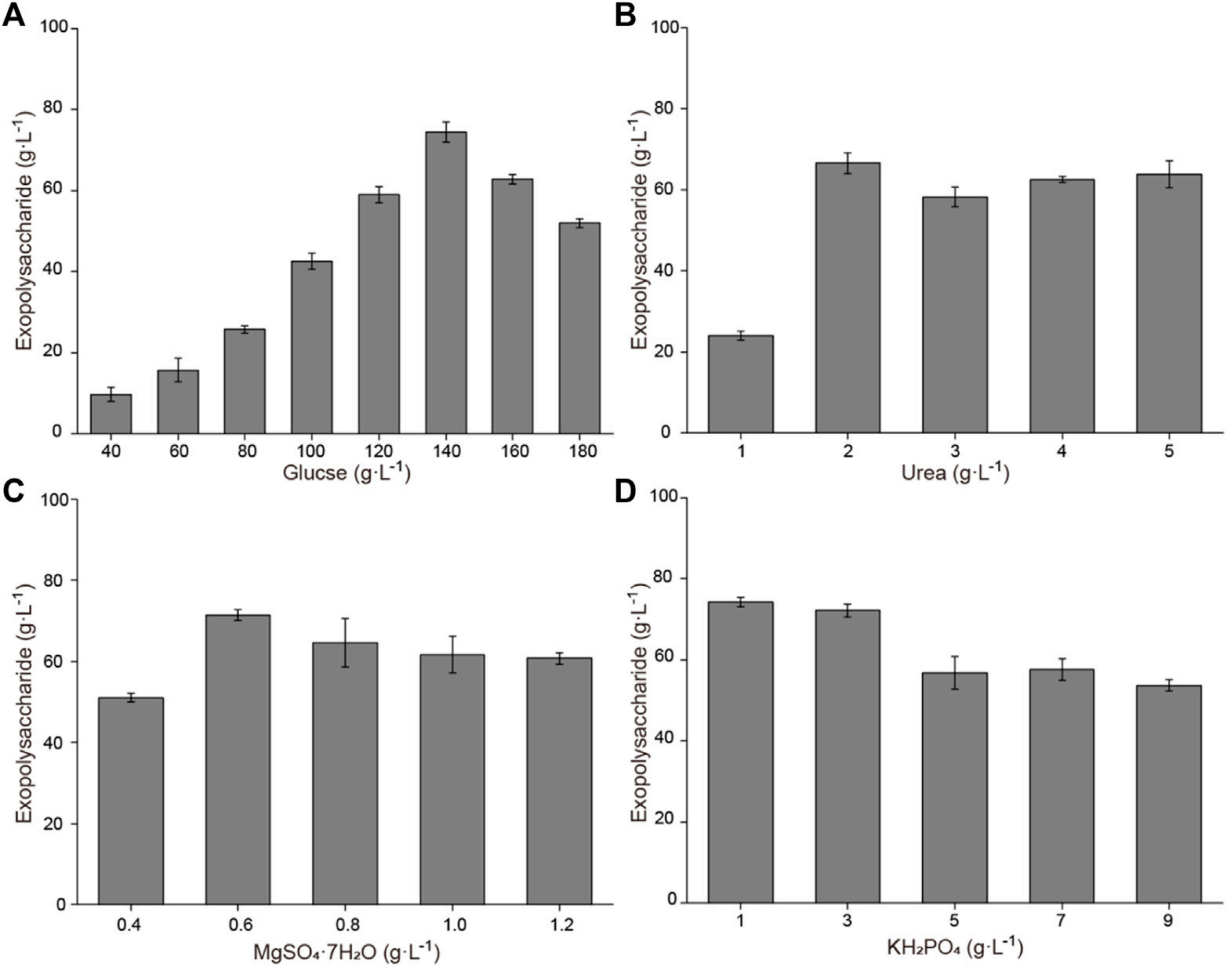
Effects of medium components on exopolysaccharide accumulation in flasks. **(A)** Effects of glucose concentration on exopolysaccharide accumulation. **(B)** Effects of urea concentration on exopolysaccharide accumulation. **(C)** Effects of MgSO_4_·7H_2_O concentration on exopolysaccharide accumulation. **(D)** Effects of KH_2_PO_4_ concentration on exopolysaccharide accumulation.

Based on the previously obtained initial fermentation medium, the influence of different medium components (glucose, urea, MgSO_4_·7H_2_O, and KH_2_PO_4_) on exopolysaccharide accumulation is shown in Figure 1. Increasing glucose concentrations result in enhanced exopolysaccharide production. When the concentration was 140 g L^−1^, the exopolysaccharide production was highest, reaching 74.4 g L^−1^. The effect of urea addition on exopolysaccharide production was not significant; the production was highest when the concentration was 2 g L^−1^. A relatively high exopolysaccharide production was obtained when the MgSO_4_·7H_2_O concentration was 0.6 g L^−1^. A high exopolysaccharide production could be obtained when the KH_2_PO_4_ concentration was 1 g L^−1^ or 3 g L^−1^, while the exopolysaccharide production decreased when the KH_2_PO_4_ concentration was further increased. To obtain the optimal combination, an orthogonal experiment for addition of glucose, urea, MgSO_4_·7H_2_O, and KH_2_PO_4_ was designed. Results showed that 89.7 g L^−1^ of exopolysaccharide was harvested with a composition of 150 g L^−1^ of glucose, 5 g L^−1^ of urea, 0.9 g L^−1^ of MgSO_4_·7H_2_O, and 3 g L^−1^ of KH_2_PO_4_ (Table S1). Statistical analysis showed that the effect of the initial glucose concentration on exopolysaccharide accumulation is extremely significant (Table S2).

### Effects of agitation speed on exopolysaccharide accumulation

**FIGURE 2:**
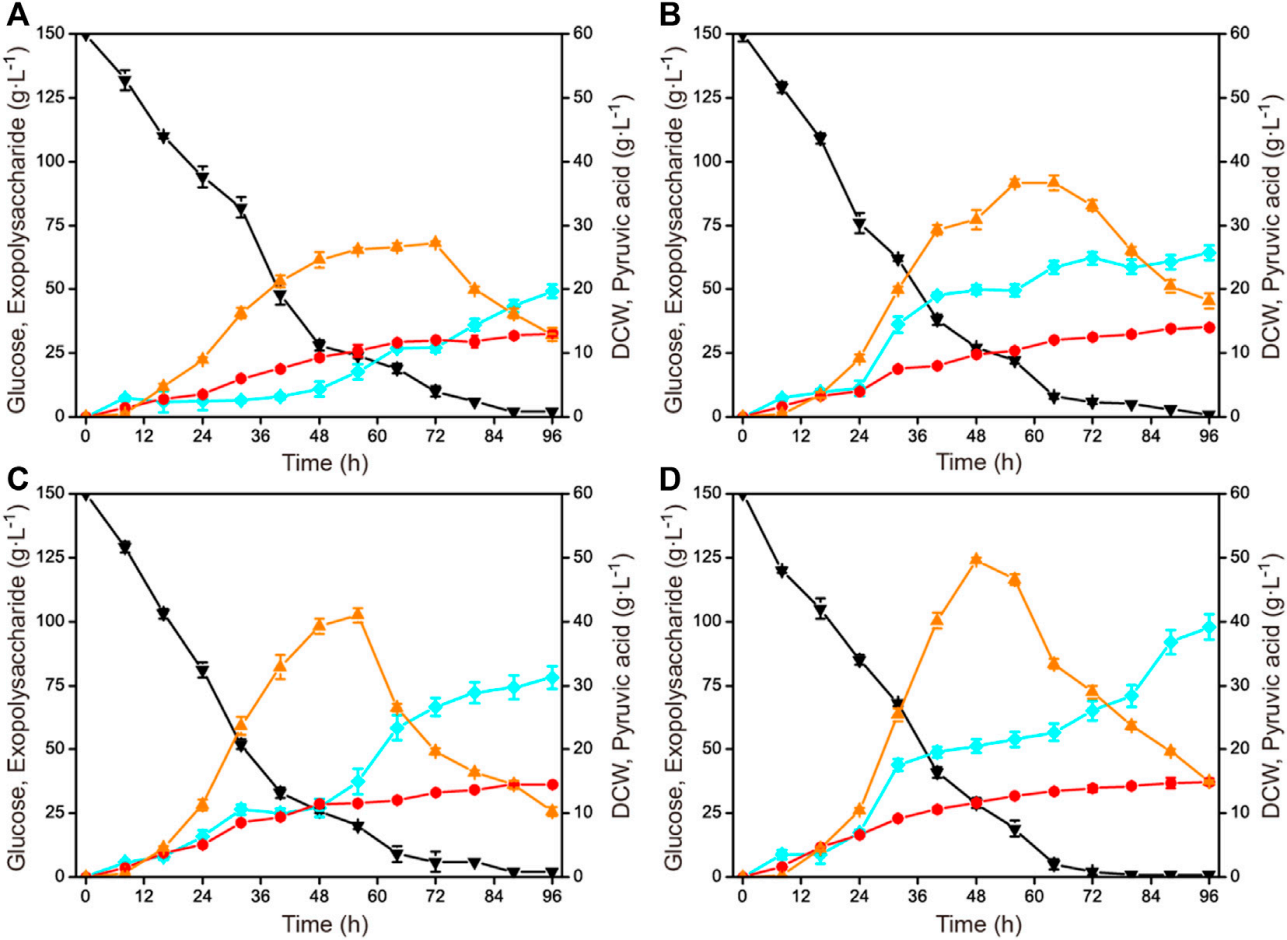
Time courses of exopolysaccharide production at different agitation speeds in a 1-L fermenter. **(A)** 1.0 vvm and 300 rmin^−1^. **(B)** 1.0 vvm and 400 rmin^−1^. **(C)** 1.0 vvm and 500 rmin^−1^. **(D)** 1.0 vvm and 600 rmin^−1^. Black down triangles, glucose; orange up triangles, pyruvic acid; red circles, DCW; blue squares, exopolysaccharide.

To enhance the exopolysaccharide production, the cultivation condition was further optimized based on obtaining the optimal fermentation medium compositions. The influences of different agitation speeds (300 rmin^−1^, 400 rmin^−1^, 500 rmin^−1^, and 600 rmin^−1^) on exopolysaccharide production in a 1-L fermenter are shown in Figure 2. With the increase of agitation speed, the biomass and the exopolysaccharide production improved. The highest exopolysaccharide production was obtained when the agitation speed was 600 rmin^−1^, reaching 97.9 g L^−1^ with a glucose yield of 0.65 g g^−1^. In addition, the time profiles of the by-product pyruvic acid accumulation showed the same trend under different agitation speeds. The accumulation gradually increased and then decreased, but the fermentation time for pyruvic acid to peak value gradually shortened.

Agitation speed is an important parameter affecting the dissolved oxygen level during the fermentation process (Huang et al., 2020; Fang et al., 2021). Considering the cultivation mode without pressure on the glass fermenter, it is generally preferred to optimize the agitation speed. There have been many examples of improving the accumulation of specific target products by systematically optimizing the agitation speed. By establishing a two-stage agitation speed controlling strategy, the production and molecular weight of pullulan with *Aureobasidium pullulans* were simultaneously enhanced (Wang D. H. et al., 2016). By studying the influence of the agitation speed, an optimal agitation speed was obtained for 1,3-propanediol production with *Shimwellia blattae* (Rodriguez et al., 2016). *C. glabrata* has been proved to require a relatively high agitation speed for pyruvic acid production (Luo et al., 2018; Guo et al., 2020b). The results obtained in this study indicated that a higher stirring speed was also beneficial to the accumulation of polysaccharides.

### Effects of aeration rate on exopolysaccharide accumulation

**FIGURE 3:**
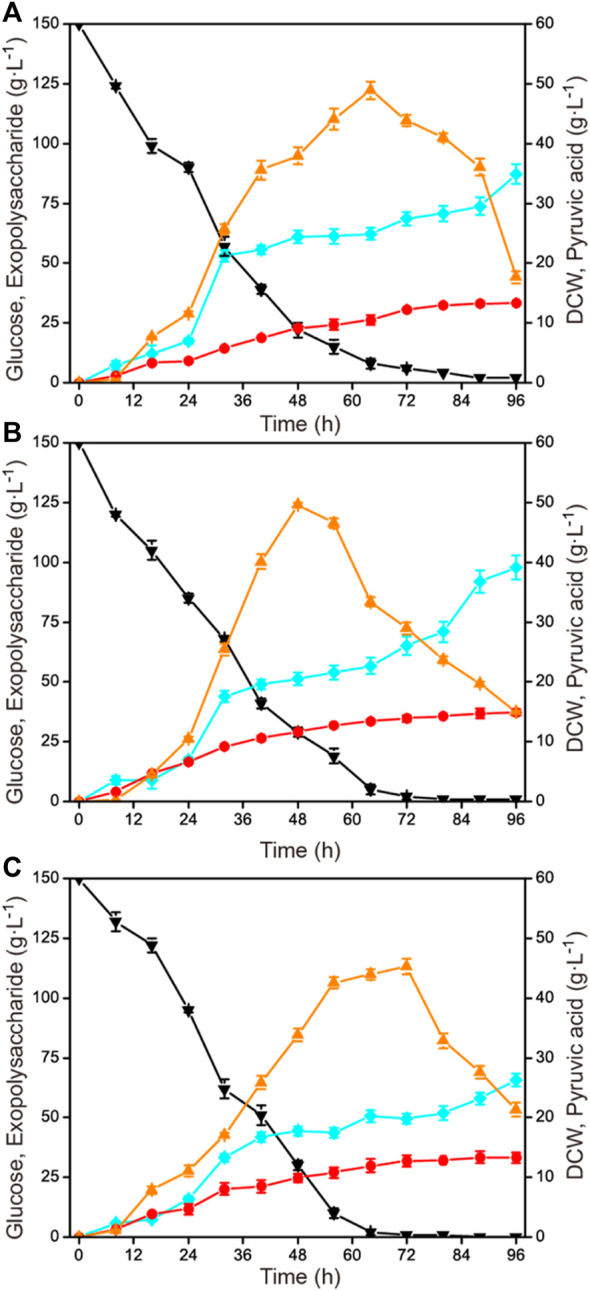
Time courses of exopolysaccharide production at different aeration rates in a 1-L fermenter. **(A)** 600 rmin^−1^ and 0.5 vvm. **(B)** 600 min^−1^ and 1.0 vvm. **(C)** 600 rmin^−1^ and 1.5 vvm. Black down triangles, glucose; orange up triangles, pyruvic acid; red circles, DCW; blue squares, exopolysaccharide.

To investigate the influence of aeration rate on exopolysaccharide accumulation, three different aeration rates (0.5 vvm, 1.0 vvm, and 1.5 vvm) were selected. The results are shown in Figure 3. When the aeration rate was controlled at a relatively low value (0.5 vvm), the growth rate and the glucose consumption rate were obviously lower than under the other two conditions (1.0 vvm and 1.5 vvm). When the aeration rate was 1.5 vvm, the highest exopolysaccharide production was just 65.8 g L^−1^, which was significantly lower than at 0.5 vvm and 1.0 vvm. Exopolysaccharide yields of 87.2 g L^−1^ and 97.9 g L^−1^ were obtained under 0.5 vvm and 1.0 vvm, respectively.

Aeration rate is another important parameter affecting the dissolved oxygen level during the fermentation process. In many fermentation processes, the supply of oxygen plays a crucial role in the growth of cells and the synthesis of specific target compounds (Alonso et al., 2012; Fang et al., 2021). Generally, the optimization of aeration rate is combined with the agitation speed for co-regulating dissolved oxygen levels. A series of combinatorial optimization strategies have been applied for the synthesis of some important products (Wang et al., 2010; Song et al., 2018). A high dissolved oxygen level could promote cell growth and pyruvic acid production at the expense of glucose consumption in *C. glabrata*, and a strategy has been used to reduce the dependence of cells on oxygen (Luo et al., 2019; Luo et al., 2020). Notably, a high aeration rate was detrimental to exopolysaccharide production, as shown in Figure 3.

### Enhancement of exopolysaccharide production by fed-batch strategy

**FIGURE 4:**
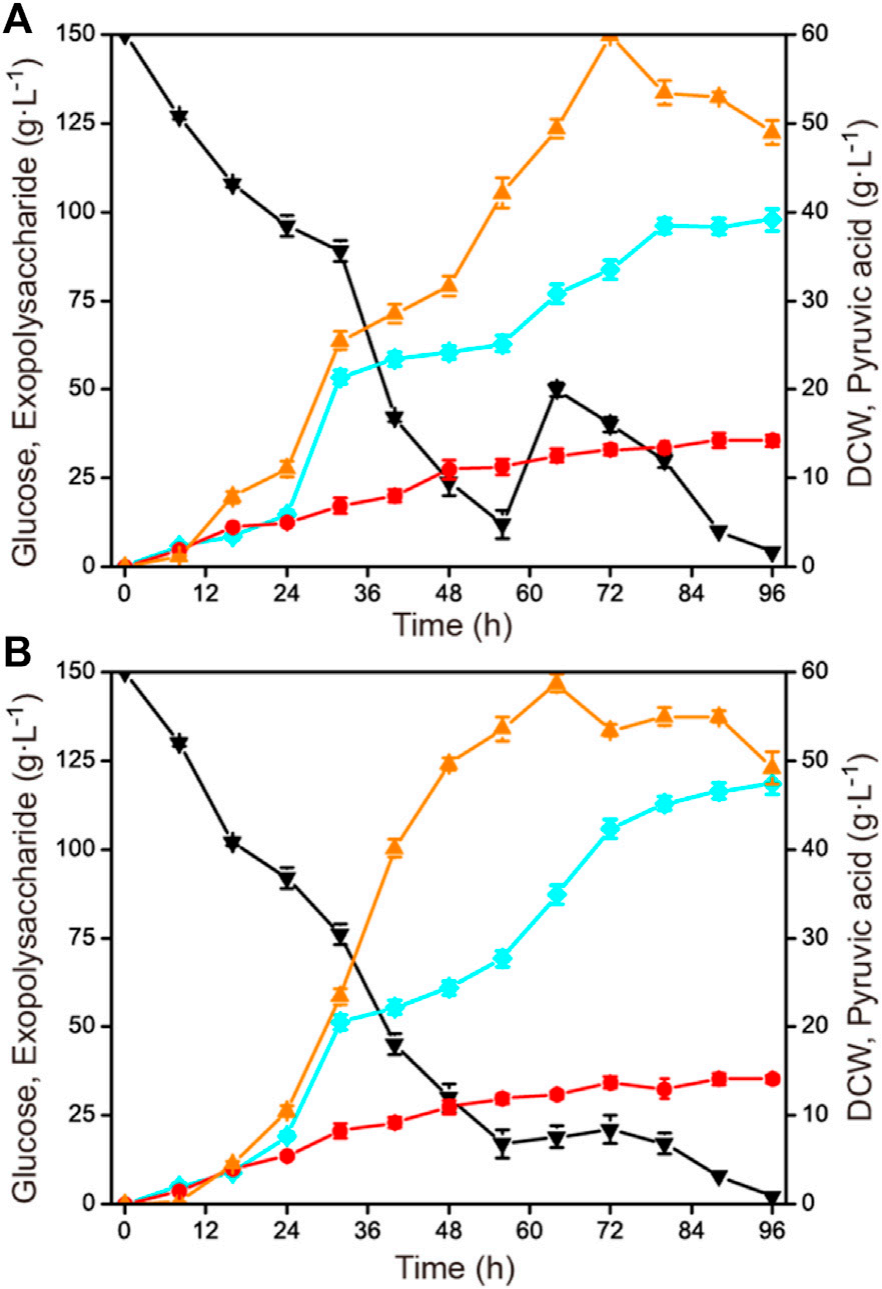
Effects of different feeding strategies on exopolysaccharide production in a 1-L fermenter. The initial concentration of glucose was 150 g L^−1^, and then 40 g L^−1^ of glucose was fed with different feeding strategies. **(A)** One-dose feeding fermentation mode of 40 g L^−1^ glucose at 56 h. **(B)** Constant rate feeding fermentation mode of 40 g L^−1^ glucose during 56–84 h. Black down triangles, glucose; orange up triangles, pyruvic acid; red circles, DCW; blue squares, exopolysaccharide.

After the optimization of agitation speed and aeration rate in the 1-L fermenter, feeding strategies were tried to further improve the accumulation of exopolysaccharide, such as single-dose fed-batch mode and constant rate feeding mode. The initial concentration of glucose was 150 g L^−1^ and the total concentration was 190 g L^−1^. When the residual glucose concentration was reduced to 15–20 g L^−1^, an additional 40 g L^−1^ of glucose was fed by a single dose at 56 h and by a constant rate of 1.43 g L^−1^ h^−1^ during 56–84 h, respectively (Figure 4). It was shown that the constant rate feeding mode yielded a better result, with a highest exopolysaccharide production of 118.6 g L^−1^, a glucose yield of 0.62 g g^−1^, and a productivity of 1.24 g L^−1^ h^−1^, which were all higher than those of by the single-dose fed-batch mode (with a highest exopolysaccharide production of 97.7 g L^−1^, a glucose yield of 0.51 g g^−1^, and a productivity of 1.02 g L^−1^ h^−1^).

The fed-batch fermentation mode is a common fermentation strategy in the field of microbial industrial fermentation. It can be divided into a variety of feeding modes, including single-dose fed-batch mode, intermittent fed-batch mode, constant rate feeding mode, exponential feeding mode, etc. (Zeng W. Z. et al., 2019; Wang et al., 2020; de Oliveira et al., 2021). These established feeding modes have been widely applied to improve the production, yield, and productivity of some specific target products, such as 2-phenylethanol, scleroglucan, and keto acids (Zeng et al., 2017; Tan et al., 2019; Tian et al., 2020). However, it should be noted that the optimal feeding mode for industrial microorganisms to produce diverse metabolites is different. In this study, the constant rate feeding mode was proved more suitable for exopolysaccharide production in *C. glabrata*, but the fed-batch strategy could be optimized more detailedly in the future, for example by comparing other feeding modes or combining with other strategies in the fed-batch process.

### Scaling up of exopolysaccharide production in a 15-L fermenter

**FIGURE 5:**
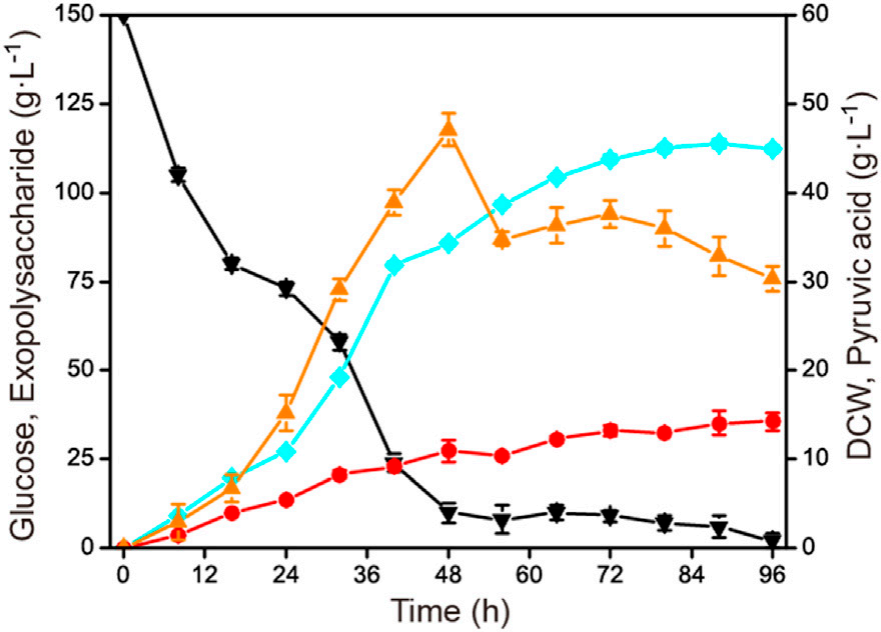
Time course of exopolysaccharide accumulation in a 15-L fermenter. The initial concentration of glucose was 150 g L^−1^, and then 40 g L^−1^ glucose was fed constantly during 56–84 h. Fermentation was conducted at 30°C, 500 rmin^−1^, and 1.0 vvm for 96 h. Black down triangles, glucose; orange up triangles, pyruvic acid; red circles, DCW; blue squares, exopolysaccharide.

In order to verify the constant rate feeding mode under the conditions that were closest to industrial production, the established process was further scaled up to a 15-L fermenter. As in the 1-L fermenter, the aeration rate was controlled at 1.0 vvm and 40 g L^−1^ was fed with a constant rate of 1.43 g L^−1^ h^−1^ during 56–84 h. The agitation speed was maintained at 500 rmin^−1^ under a fermenter pressure of 0.035 MPa. The result is shown in Figure 5. The highest exopolysaccharide titer was obtained at 88 h, reaching 113.8 g L^−1^ with a glucose yield of 0.60 g g^−1^ and a productivity of 1.29 g L^−1^ h^−1^. Compared with the constant rate feeding mode in the 1-L fermenter, the exopolysaccharide production was slightly decreased. Additionally, during the fermentation process in the 15-L fermenter, the highest and the final accumulation of by-product pyruvic acid were also obviously less than in the 1-L fermenter. The specific fermentation characteristics in 1-L and 15-L fermenters are presented in Table 1.

**TABLE 1:** Fermentation characteristics in 1-L and 15-L fermenters.

**Fermentation characteristics**	**Bach fermentation (1 L)**	**Constant speed feeding fermentation (1 L)**	**Constant speed feeding fermentation (15 L)**
Time (h)	96	96	88
Glucose consumption (g·L^−1^)	150	190	190
DCW (g·L^−1^)	14.9	14.1	14.0
Exopolysaccharide (g·L^−1^)	97.9	118.6	113.8
Yield of exopolysaccharide (g·g^−1^)	0.65	0.62	0.60
Productivity of exopolysaccharide (g·L^−1^ h^−1^)	1.02	1.24	1.29
Pyruvic acid (g·L^−1^)	14.4	49.3	32.8

The development of commercial production methods of a specific product with microorganism fermentation is a long amplification process, from microtiter plates, to shaking flasks, to bench-scale fermenters, to pilot-scale fermenters, and ultimately to commercial fermenters (Wehrs et al., 2019; Ganeshan et al., 2021). The scaling up of the fermentation process for a target product is a systematic project, but not simply a matter of increasing cultivation and vessel volume (Hewitt & Nienow, 2007; Krajang et al., 2021). A reliable scaling up process at the lab scale should reasonably solve the problem from shaking flasks to bench-scale fermenters, particularly the fermenters equipped with pressure control. A series of scaling up processes has been published for some exopolysaccharides, such as glucan, xanthan gum, levan, and pullulan (Freitas et al., 2017; Bzducha-Wrobel et al., 2018; Vaishnav et al., 2022). In this study, the exopolysaccharide production after scaling up to a 15-L fermenter was slightly decreased. But the time to reach the maximum was shortened, thereby achieved a relatively higher space time yield. To further enhance the production, the feeding rate and the glucose concentration could be appropriately increased in the fed-batch process.

## Conclusion

In this study, the fermentation medium components were optimized in shaking flasks to enhance exopolysaccharide production with *C. glabrata* strain 4-C10, which was obtained by random mutagenesis. By optimizing the agitation speed and aeration rate and establishing a constant rate feeding fermentation mode in a 1-L fermenter, the exopolysaccharide titer was enhanced to 118.6 g L^−1^, with a glucose yield of 0.62 g g^−1^ and a productivity of 1.24 g L^−1^ h^−1^. After scaling up to a 15-L fermenter, the production was slightly decreased, reaching 113.8 g L^−1^. The obtained results could provide references for industrial exopolysaccharide production.

## Data Availability

The original contributions presented in the study are included in the article/Supplementary Material, further inquiries can be directed to the corresponding author.
