# Supplementary material for: Efficient biosynthesis of exopolysaccharide in Candida glabrata by a fed-batch culture
Source: Front Bioeng Biotechnol. 2022 Sep 2;10:987796. doi: 10.3389/fbioe.2022.987796 (PMC9478339; doi:10.3389/fbioe.2022.987796)
Supplement: Supplementary file 1 [file Table1.DOCX]

## Table S1 Results of orthogonal experimental design.

| **No.** | **Glucose (g·L^−1^)** | **Urea**  **(g·L^−1^)** | **MgSO_4_·7H_2_O (g·L^−1^)** | **KH_2_PO_4_ (g·L^−1^)** | **Exopolysaccharide (g·L^−1^)** |
| --- | --- | --- | --- | --- | --- |
| 1 | 135 | 3.5 | 1.2 | 5 | 38.4 |
| 2 | 150 | 3.5 | 0.9 | 5 | 59.2 |
| 3 | 135 | 3.5 | 0.6 | 1 | 45.9 |
| 4 | 120 | 3.5 | 1.2 | 3 | 31.2 |
| 5 | 120 | 5 | 0.9 | 3 | 41.9 |
| 6 | 135 | 2 | 0.9 | 5 | 49.8 |
| 7 | 135 | 3.5 | 0.6 | 5 | 36.9 |
| 8 | 150 | 3.5 | 1.2 | 3 | 53.2 |
| 9 | 135 | 5 | 0.6 | 3 | 45.7 |
| 10 | 120 | 3.5 | 0.6 | 3 | 56.4 |
| 11 | 135 | 5 | 1.2 | 3 | 68.0 |
| 12 | 120 | 2 | 0.9 | 3 | 77.2 |
| 13 | 150 | 5 | 0.9 | 3 | 89.7 |
| 14 | 120 | 3.5 | 0.9 | 1 | 56.8 |
| 15 | 135 | 2 | 0.9 | 1 | 69.6 |
| 16 | 120 | 3.5 | 0.9 | 5 | 56.2 |
| 17 | 150 | 2 | 0.9 | 3 | 77.3 |
| 18 | 135 | 5 | 0.9 | 5 | 62.1 |
| 19 | 135 | 3.5 | 1.2 | 1 | 63.3 |
| 20 | 135 | 3.5 | 0.9 | 3 | 85.2 |
| 21 | 150 | 3.5 | 0.6 | 3 | 80.0 |
| 22 | 135 | 3.5 | 0.9 | 3 | 70.7 |
| 23 | 135 | 2 | 0.6 | 3 | 66.9 |
| 24 | 135 | 5 | 0.9 | 1 | 60.8 |
| 25 | 150 | 3.5 | 0.9 | 1 | 73.7 |
| 26 | 135 | 2 | 1.2 | 3 | 69.8 |
