# Supplementary material for: Efficient biosynthesis of exopolysaccharide in Candida glabrata by a fed-batch culture
Source: Front Bioeng Biotechnol. 2022 Sep 2;10:987796. doi: 10.3389/fbioe.2022.987796 (PMC9478339; doi:10.3389/fbioe.2022.987796)
Supplement: Supplementary file 2 [file Table2.DOCX]

## Table S2 Statistical analysis of orthogonal experiment results.

| **Factor** | **Low level** | **High Level** | **F value** | **P value** | **Significant Difference** |
| --- | --- | --- | --- | --- | --- |
| Glucose | 120 | 150 | 10.48 | 0.0060 | ** |
| Urea | 2 | 5 | 1.46 | 0.2476 |  |
| MgSO_4_·7H_2_O | 0.6 | 1.2 | 0.0509 | 0.8248 |  |
| KH_2_PO_4_ | 1 | 5 | 3.61 | 0.0781 |  |

***P* < 0.01.
